# Assessment of the Prognostic Performance of the Oakland Score in Lower Gastrointestinal Bleeding: A Retrospective Cohort Study

**DOI:** 10.3390/diagnostics16142150

**Published:** 2026-07-09

**Authors:** Sebnem Sahan, Mehmet Emin Arayici, Goksel Bengi, Süleyman Dolu

**Affiliations:** 1 Department of Internal Medicine, Faculty of Medicine, Dokuz Eylül University, 35340 İzmir, Türkiye; 2 Department of Biostatistics and Medical Informatics, Faculty of Medicine, Dokuz Eylül University, 35340 İzmir, Türkiye; mehmet.e.arayici@gmail.com; 3 Department of Gastroenterology, Faculty of Medicine, Dokuz Eylül University, 35340 İzmir, Türkiye

**Keywords:** Oakland Score, lower gastrointestinal bleeding, prognosis, risk stratification

## Abstract

**Background/Objectives:** Lower gastrointestinal bleeding (LGIB) is a common and potentially life-threatening emergency that disproportionately affects elderly, comorbid patients, yet evidence-based risk stratification tools remain underused. The Oakland Score was developed to identify patients who can be safely discharged. This study aimed to determine prognostic factors in patients presenting with LGIB and to evaluate the relationship between the Oakland Score and adverse clinical outcomes. **Methods:** In this single-centre, retrospective, descriptive study, patients aged 18 years and older who presented to the emergency department with LGIB between 2015 and 2024, and who were evaluated, treated and followed up by the Department of Gastroenterology at Dokuz Eylül University, and who underwent endoscopic evaluation were reviewed. A total of 890 patients who met the inclusion criteria and had complete medical records, defined as full availability of all Oakland Score variables and primary outcome data, were included in the final analysis. The Oakland Score was calculated for every patient, and its association with mortality, intensive care unit (ICU) admission, blood-product transfusion, early and late rebleeding, and the need for emergency surgery was analysed. Receiver operating characteristic (ROC) analysis was used to assess discriminative performance. **Results:** The mean age was 69.8 ± 15.6 years, and 50.4% of patients were female. The most frequent comorbidities were hypertension (56.2%), coronary artery disease (28.9%) and diabetes mellitus (25.1%). Diverticular bleeding was the most common aetiology (25.1%). Red blood cell transfusion was required in 52.8% of patients, and the in-hospital mortality rate was 6.1%. The Oakland Score was significantly associated with mortality, ICU admission, blood-product transfusion, early and late rebleeding, and emergency surgery (all *p* < 0.05). On ROC analysis the score performed best for ICU admission (AUC 0.754) and mortality (AUC 0.706), and was significantly associated with red blood cell, platelet and fresh frozen plasma transfusion requirements (*p* < 0.001). On multivariable logistic regression, the Oakland Score was an independent predictor of one-month rebleeding (OR 1.082; 95% CI 1.032–1.133; *p* = 0.001) but did not retain independent significance for mortality or ICU admission after adjustment for malignancy, serum albumin and BUN. **Conclusions:** The Oakland Score is significantly associated with major adverse outcomes in LGIB and is particularly sensitive for predicting mortality and ICU admission. It is a useful adjunct to clinical judgement for risk stratification, although it should be interpreted alongside the patient’s overall clinical status.

## 1. Introduction

Lower gastrointestinal bleeding (LGIB) refers to the abrupt loss of blood originating from intestinal segments distal to the ligament of Treitz. Acute LGIB accounts for approximately 20% of all gastrointestinal haemorrhages, and more than half of these cases are clinically severe enough to require hospital admission [1,2]. A substantial proportion of patients presenting with acute LGIB are elderly and frequently carry multiple comorbid conditions such as myocardial infarction, diabetes mellitus and malignant neoplasia [3,4,5]. Although most patients achieve clinical stability with conservative management, a meaningful minority require inpatient care, including hospitalisation and red blood cell transfusion.

Randomised controlled trials addressing the management of LGIB are limited in both number and scope compared with those for acute upper gastrointestinal bleeding. This relative paucity of high-quality evidence translates into uncertainty in clinical decision-making regarding risk classification, the optimal timing of endoscopy, target haemoglobin thresholds, and the selection of appropriate haemostatic interventions [2,3,4].

Diverticular bleeding is the single most common cause of LGIB, accounting for more than 20% of hospitalised cases, followed by anorectal disease [6,7,8]. Haemorrhoidal bleeding is identified in roughly 12–21% of patients admitted for LGIB and is typically low-volume and self-limiting [9,10,11]. Other recognised aetiologies include ischaemic and other forms of colitis, radiation proctitis, post-polypectomy iatrogenic bleeding, vascular anomalies such as angioectasia, and colorectal malignancy. In a notable subset of patients, no specific source is identified despite thorough evaluation; recent estimates suggest that no definite aetiology is established in approximately one in five patients with acute LGIB [2,7].

Effective management of LGIB depends on accurate risk assessment and prognostic estimation. The Oakland Score, originally developed and validated in 2017 in patients hospitalised for LGIB in the United Kingdom [1], is a risk stratification instrument designed to support safe-discharge decisions and to optimise the use of inpatient resources. The score comprises seven variables—age, sex, previous admission for LGIB, findings on digital rectal examination, heart rate, systolic blood pressure and haemoglobin concentration—and ranges from 0 to 35, with higher values indicating a greater probability of an adverse outcome.

The principal aim of the present study was to comprehensively examine the relationship between the Oakland Score and prognosis in patients with LGIB, and to clarify how the score might be more effectively integrated into clinical practice. To this end, we sought to evaluate the reliability of the Oakland Score, its role across the treatment pathway, and the manner in which it relates to clinically important outcomes.

## 2. Materials and Methods

### 2.1. Ethical Approval

The study was conducted following approval by the Non-Interventional Research Ethics Committee of Dokuz Eylül University (decision dated 14 May 2025; file number 9387-GOA).

### 2.2. Study Design and Population

This retrospective, descriptive study was performed at the Department of Gastroenterology, Faculty of Medicine, Dokuz Eylül University, covering the period from 2015 to 2024. The study population comprised patients who presented to the emergency department with LGIB and whose diagnosis, treatment and follow-up were carried out at our clinic. Inclusion criteria were age 18 years or older, presentation with LGIB, endoscopic evaluation by colonoscopy or flexible sigmoidoscopy, and completion of follow-up at our institution. Exclusion criteria were age younger than 18 years, absence of endoscopic evaluation, and inaccessible or incomplete medical records. On this basis, a total of 890 patients were enrolled. Completeness of records was defined as the availability of all variables required for Oakland Score calculation (age, sex, prior LGIB-related hospital admission, heart rate, systolic blood pressure and pre-colonoscopy haemoglobin), together with full documentation of presenting symptoms, comorbidities, medication history, endoscopic findings and primary outcome data (mortality, ICU admission, rebleeding, transfusion requirements and surgical intervention). All 890 included patients had complete data for these variables.

### 2.3. Data Collection and Variables

Demographic characteristics (age, sex), presenting symptoms (e.g., syncope/pre-syncope), comorbidities (hypertension, diabetes mellitus, coronary artery disease, cerebrovascular accident, inflammatory bowel disease, malignancy and cirrhosis) and regularly used medications (acetylsalicylic acid, clopidogrel, warfarin, direct oral anticoagulants, low-molecular-weight heparin, non-steroidal anti-inflammatory drugs and steroids) were recorded retrospectively from the hospital information management system and patient files. Any prior episode of LGIB or related hospital admission was also documented.

### 2.4. Endoscopic Findings, and Laboratory and Clinical Data

All patients underwent colonoscopy or flexible sigmoidoscopy. Bleeding aetiologies were categorised as diverticular, angiodysplasia, neoplasia (polyp and tumour), inflammatory causes (ulcerative colitis, Crohn’s disease, infection, ischaemic colitis, proctitis, non-specific lesions), anorectal disease (haemorrhoids, fissure, rectal ulcer), post-polypectomy bleeding, anastomotic-line bleeding and other causes. When endoscopic therapy was applied, argon plasma coagulation (APC), haemoclip placement and adrenaline injection were the preferred methods. Recorded laboratory parameters included the lowest pre-colonoscopy haemoglobin, the lowest haemoglobin within the first 24 h, platelet count, white blood cell (WBC) count, neutrophils, mean platelet volume (MPV), red cell distribution width (RDW), blood urea nitrogen (BUN), creatinine, albumin, lactate dehydrogenase (LDH) and the international normalised ratio (INR). Vital signs (heart rate, systolic and diastolic blood pressure) and the Oakland Score were obtained for every patient. The Oakland Score was calculated using the haemoglobin concentration obtained at the time of the patient’s initial presentation to the emergency department (admission haemoglobin), together with the other variables included in the original Oakland Score (age, sex, previous LGIB admission, digital rectal examination findings, heart rate, and systolic blood pressure) [1,2].

### 2.5. Clinical Outcomes

Prognostic endpoints comprised admission status (ward/ICU), length of stay, mode of discharge, blood-product replacement requirements (red blood cells, platelets, fresh frozen plasma), rebleeding within one month and within one year, the need for elective or emergency surgery, and mortality. The relationship between the Oakland Score and each of these parameters was examined.

### 2.6. Statistical Analysis

Statistical analyses were performed using IBM SPSS Statistics, version 30.0 (IBM Corp., Armonk, NY, USA). The normality of continuous variables was assessed using the Kolmogorov–Smirnov test and visual inspection of histograms. Normally distributed continuous variables are presented as mean ± standard deviation (SD); non-normally distributed variables as median with minimum–maximum range; and categorical variables as frequency and percentage. Comparisons of continuous variables between two independent groups were performed using the independent-samples Student’s *t*-test for normally distributed data and the Mann–Whitney U test for non-normally distributed data. Differences in categorical variables were evaluated using Pearson’s chi-square test, the Yates-corrected chi-square test for 2 × 2 tables with small expected frequencies, or Fisher’s exact test, as appropriate. For comparisons across three or more independent groups, one-way analysis of variance (ANOVA) with Bonferroni post hoc correction was applied for normally distributed data, and the Kruskal–Wallis test for non-normally distributed data. The prognostic performance of the Oakland Score and selected laboratory parameters was assessed by receiver operating characteristic (ROC) analysis. The area under the ROC curve (AUC) was used as the measure of discriminative ability and was interpreted as follows: AUC near 0.5, non-informative; 0.7–0.8, acceptable; 0.8–0.9, good; ≥0.9, excellent. Optimal cut-off values were determined by the Youden index (sensitivity + specificity − 1), and the corresponding sensitivity and specificity are reported. Ninety-five percent confidence intervals (95% CIs) for AUC values were calculated using the DeLong method. ROC curves with 95% confidence bands were generated using the binormal model. All figures were produced with Python 3.10.12 using the Matplotlib (version 3.10.9) and SciPy (version 1.15.3) libraries. To assess the independent prognostic value of the Oakland Score and other clinical factors, multivariable binary logistic regression was performed for each primary outcome (mortality, ICU admission, one-month rebleeding and emergency surgery). Because the Oakland Score already encodes age, sex, heart rate, systolic blood pressure, haemoglobin and prior LGIB admission, models were constructed using the Oakland Score together with clinically relevant variables not contained in the score, thereby avoiding multicollinearity. Variables entered simultaneously into each model were selected on the basis of clinical relevance and univariate significance. Results are expressed as odds ratios (ORs) with 95% confidence intervals (CIs). A two-tailed *p* value of < 0.05 was considered statistically significant.

## 3. Results

### 3.1. Patient Characteristics and Demographics

Among the 890 patients included, the mean age was 69.8 ± 15.6 years (range 18–102 years). Of these, 449 (50.4%) were female and 441 (49.6%) male, and 47 patients (5.3%) presented with syncope or pre-syncope. The most common comorbidity was hypertension (56.2%), followed by coronary artery disease (28.9%), diabetes mellitus (25.1%), malignancy (22.8%), cerebrovascular accident (9.2%), inflammatory bowel disease (4.2%) and cirrhosis (3.4%). With respect to medication use, acetylsalicylic acid was the most frequently used agent (28.9%), followed by clopidogrel (13.6%), non-steroidal anti-inflammatory drugs (12.7%), direct oral anticoagulants (9.2%), warfarin (8.1%), steroids (6.3%) and low-molecular-weight heparin (5.6%) (Table 1).

### 3.2. Laboratory, Vital and Oakland Score Data

The mean lowest pre-colonoscopy haemoglobin was 8.95 ± 2.52 g/dL and the mean lowest haemoglobin within the first 24 h was 9.16 ± 2.56 g/dL. The mean platelet count was 242 ± 113.7 × 10^3^/µL and the mean WBC count 9.29 ± 4.95 × 10^3^/µL. Mean BUN was 29 ± 21.76 mg/dL, mean creatinine 1.19 ± 0.9 mg/dL, and mean albumin 3.34 ± 0.6 g/dL. The mean heart rate was 87.8 ± 17 beats/min, the mean systolic blood pressure 127.3 ± 24 mmHg, and the mean diastolic blood pressure 75.4 ± 15.2 mmHg. The mean Oakland Score was 22.28 ± 6.15 (Table 2).

### 3.3. Endoscopic Diagnosis and Treatment

The most common cause of LGIB was diverticular disease (25.1%), followed by inflammatory conditions (21.2%), neoplasia (19.9%), anorectal disease (13.9%), angiodysplasia (11.0%), other causes (4.4%), post-polypectomy bleeding (2.6%) and anastomotic bleeding (1.8%) (Table 3). The majority of patients (80.4%) received no endoscopic therapy; among those treated, APC was most frequently used (11.9%), followed by haemoclip placement (5.4%) and adrenaline injection (2.2%). A previous episode of LGIB was reported by 26.4% of patients and a prior LGIB-related admission by 12.5%. The median ward stay was 7 days (range 1–61) and the median ICU stay was 4 days (range 2–46).

### 3.4. Prognostic Outcomes

Red blood cell (RBC) transfusion was required in 52.8% of patients; of these, 60% received three units or fewer and 40% received more than three units. Fresh frozen plasma (FFP) was required by 17.75% and platelet transfusion by 4.61%. Rebleeding occurred in 7.2% of patients within the first month and in 18.2% within the first year. Hospital admission was required in 70.2% of patients, ICU admission in 4.15%, surgery in 10.0% and emergency surgery in 4.8%. The overall in-hospital mortality rate was 6.1% (Table 4).

### 3.5. Factors Associated with Mortality

Patients who died were significantly older than survivors (75.6 ± 12.8 vs. 69.5 ± 15.7 years; *p* = 0.005). Sex was not associated with mortality (*p* = 0.441). Among comorbidities, malignancy (15.3% vs. 3.4%; *p* < 0.001) and cirrhosis (16.7% vs. 5.7%; *p* = 0.030) were significantly associated with death, whereas hypertension, diabetes, coronary artery disease, cerebrovascular accident and inflammatory bowel disease were not. With regard to medication, low-molecular-weight heparin (16% vs. 5.5%; *p* = 0.008) and steroid use (17.9% vs. 5.3%; *p* = 0.001) were significantly associated with mortality. Higher heart rate and lower systolic and diastolic blood pressure were also significantly associated with death (all *p* ≤ 0.003). Lower haemoglobin, lower platelet count, higher WBC and neutrophil counts, higher RDW, higher BUN and creatinine, lower albumin, higher LDH and higher INR were all significantly associated with mortality (Table 5). The mean Oakland Score was significantly higher in patients who died than in survivors (26.15 ± 5.03 vs. 22.03 ± 6.13; *p* < 0.001).

### 3.6. Rebleeding, Emergency Surgery and ICU Admission

The Oakland Score was significantly higher in patients with rebleeding within one month (24.92 ± 5.53 vs. 22.08 ± 6.15; *p* < 0.001) and within one year (23.71 ± 5.68 vs. 22.09 ± 6.18; *p* = 0.011). Malignancy was significantly associated with one-month rebleeding (11.3% vs. 6.0%; *p* = 0.015), whereas hypertension, inflammatory bowel disease and cirrhosis were significantly associated with one-year rebleeding. For emergency surgery, malignancy (*p* = 0.034) and NSAID use (*p* = 0.018) were significant clinical correlates, together with higher WBC and neutrophil counts and lower albumin; the mean Oakland Score was again higher in patients requiring emergency surgery (24.42 ± 5.72 vs. 22.17 ± 6.15; *p* = 0.019). When ward, ICU and discharge groups were compared, patients admitted to the ICU had significantly higher heart rate, lower blood pressure, lower haemoglobin and albumin, higher BUN and LDH, and the highest mean Oakland Score (27.24 ± 3.66 in the ICU group vs. 23.17 ± 5.75 on the ward and 19.61 ± 6.30 among those discharged; *p* < 0.001).

### 3.7. Oakland Score and Blood-Product Replacement

The Oakland Score was strongly associated with transfusion requirements. The mean score was markedly higher among patients requiring RBC transfusion (26.57 ± 3.68 vs. 17.48 ± 4.62), platelet transfusion (27.17 ± 3.81 vs. 22.04 ± 6.14) and FFP transfusion (26.81 ± 4.42 vs. 21.30 ± 6.03); all comparisons reached statistical significance (*p* < 0.001) (Table 6).

### 3.8. ROC Analysis of the Oakland Score

**Figure 1:**
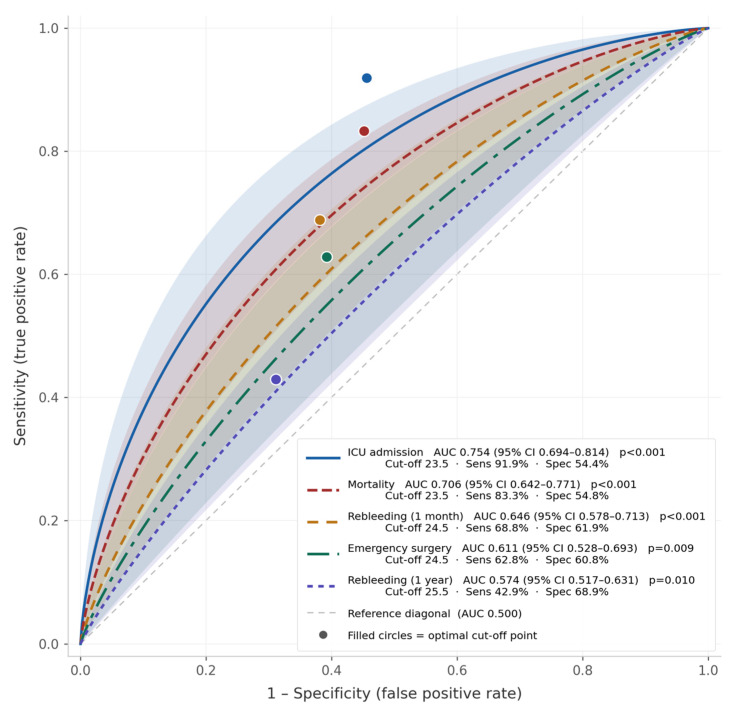
Receiver operating characteristic (ROC) curves of the Oakland Score for predicting adverse clinical outcomes in 890 patients with acute lower gastrointestinal bleeding.

ROC analysis was used to assess the performance of the Oakland Score in predicting adverse outcomes (Figure 1). The score demonstrated the highest discrimination for ICU admission (AUC 0.754; sensitivity 91.9%, specificity 54.4% at a cut-off of 23.5) and mortality (AUC 0.706; sensitivity 83.3%, specificity 54.8% at a cut-off of 23.5). Discrimination was moderate for one-month rebleeding (AUC 0.646) and emergency surgery (AUC 0.611), and weak for one-year rebleeding (AUC 0.574). All associations were statistically significant.

### 3.9. Multivariable Logistic Regression Analysis

To assess which factors independently predicted adverse outcomes, multivariable logistic regression was performed for mortality, ICU admission, one-month rebleeding and emergency surgery (Table 7). After adjustment, the Oakland Score was not independently associated with mortality (OR 1.028; 95% CI 0.954–1.108; *p* = 0.465) or ICU admission (OR 1.101; 95% CI 0.999–1.214; *p* = 0.051). The independent predictors of mortality were malignancy (OR 4.370; 95% CI 2.163–8.828; *p* < 0.001), low serum albumin (OR 0.215; 95% CI 0.109–0.422; *p* < 0.001) and elevated BUN (OR 1.018; 95% CI 1.006–1.031; *p* = 0.004). For ICU admission, independent predictors were low albumin (OR 0.177; 95% CI 0.083–0.377; *p* < 0.001), malignancy (OR 3.088; 95% CI 1.378–6.922; *p* = 0.006) and LMWH use (OR 3.991; 95% CI 1.313–12.137; *p* = 0.015). In contrast, the Oakland Score was an independent predictor of one-month rebleeding (OR 1.082 per point; 95% CI 1.032–1.133; *p* = 0.001), together with malignancy (OR 1.820; 95% CI 1.057–3.136; *p* = 0.031). For emergency surgery, independent predictors were malignancy (OR 2.212; 95% CI 1.100–4.448; *p* = 0.026), NSAID use (OR 2.871; 95% CI 1.330–6.199; *p* = 0.007) and low albumin (OR 0.482; 95% CI 0.268–0.867; *p* = 0.015); the Oakland Score was not significant in this model (*p* = 0.728). The reduced sample sizes in models including albumin (N = 708) reflect missing data for this variable (*n* = 182 missing).

## 4. Discussion

In this study of 890 patients, we evaluated the relationship between the Oakland Score and clinical prognosis in patients presenting to the emergency department with LGIB. The score was significantly associated with mortality, ICU admission, blood-product transfusion, rebleeding within one month and one year, and the need for surgery, supporting its potential value as a prognostic aid in guiding clinical decisions and patient management.

The median age of our cohort was 73 years, closely matching the median age of 74 years reported by Oakland and colleagues in the original United Kingdom cohort [1]. This reinforces the observation that LGIB is predominantly a condition of older patients. Consistent with the work of Lanas and colleagues [5], advanced age and a higher burden of comorbidity appear to be more strongly associated with LGIB than with upper gastrointestinal bleeding.

The sex distribution in our cohort (50.4% female, 49.6% male) was similar to that reported by Quach and colleagues in a multi-centre cohort (51.2% female) [6]. Some studies, by contrast, have reported a male predominance; Aoki and colleagues found that 55% of patients were male [7], Yeon and colleagues 54.5% [12] and Nagata and colleagues 61.1% [8]. Syncope or pre-syncope at presentation occurred in only 5.3% of our patients, comparable with the 6% reported by Smith and colleagues in a study of 2385 patients with acute LGIB [9].

Hypertension was the most frequent comorbidity (56.2%), followed by coronary artery disease, diabetes mellitus and malignancy, broadly consistent with the cardiovascular and metabolic comorbidity profile reported by Li and colleagues [13]. These findings likely reflect the older age of patients with LGIB and the rising prevalence of such conditions with age. Acetylsalicylic acid was the most frequently used medication (28.9%), mirroring the 28.9% reported by Aoki and colleagues among patients with severe LGIB [7], and was followed by clopidogrel and NSAIDs. The literature consistently links antiplatelet agents, NSAIDs and anticoagulants to an increased risk of LGIB; clopidogrel has been described as an independent risk factor by Delaney and colleagues [10], NSAIDs have been associated with an approximately 2.6-fold increase in risk by Hreinsson and colleagues [11], and no clear difference in LGIB risk between direct oral anticoagulants and warfarin was observed by Abraham and colleagues [14].

Diverticular bleeding was the leading aetiology (25.1%), in keeping with the established literature [7,8], followed by inflammatory bowel disease, neoplasia, anorectal disease and angiodysplasia. Most patients (80.4%) did not require endoscopic therapy, and APC was the most frequently applied technique. The relatively infrequent use of endoscopic haemostasis is consistent with the self-limiting nature of many LGIB episodes, particularly diverticular bleeding, and with the practice of performing endoscopy within the first 24–48 h of admission rather than emergently. The predominance of APC reflects its suitability for angiodysplasia and ulcer-related bleeding.

A previous episode of LGIB was reported by 26.4% of our patients, consistent with rates of 25–30% reported in other acute LGIB series [7,15]. Our rebleeding rates of 7.2% at one month and 18.2% at one year were comparable with the 4.9% and 19% reported by Aoki and colleagues [7]. The median ward stay of 7 days was similar to the median of 6 days reported by Yeon and colleagues in a Korean cohort [12] and consistent with other published series [16,17]. Our ICU admission rate of 4.1% was higher than the 0.6% reported by Devani and colleagues [18], a difference plausibly explained by the older, more comorbid case mix of our tertiary referral centre.

RBC transfusion was required in 52.8% of patients, higher than the 26.3% reported in the original Oakland cohort [1] but comparable with the 61.1% reported by Nieto and colleagues in a similarly high-acuity inpatient series [15]. The higher transfusion requirement in our cohort likely reflects the advanced age, comorbidity and the selection of patients referred specifically to gastroenterology from the emergency department. The emergency surgery rate of 4.9% was consistent with rates of 5.7% reported by Li and colleagues [13] and Smith and colleagues [9], and 6% by Camus and colleagues [19], though higher than the 2.6–3% reported in some series. Our overall mortality of 6.1% exceeded the 3.4% reported by both Smith and colleagues [9] and Radaelli and colleagues [16], again most likely because milder cases manageable in the outpatient setting were discharged without gastroenterology consultation, yielding a more severely affected study population.

Examination of vital signs and laboratory parameters showed that low systolic and diastolic blood pressure and elevated heart rate were significantly associated with ICU admission and mortality, in agreement with Laine and colleagues [20], who linked falling systolic pressure and rising heart rate to increased mortality. Lower haemoglobin and albumin and higher INR were associated with ICU admission and mortality, paralleling the adverse-outcome predictors identified by Li and colleagues [13].

Mortality in our cohort was significantly associated with advanced age, malignancy, abnormal vital signs, low haemoglobin, thrombocytopenia, elevated BUN and creatinine, high INR and low albumin, a profile consistent with that reported by Yeon and colleagues [12]; steroid use emerged as an additional correlate that warrants confirmation in larger studies.

For one-month rebleeding, malignancy, low haemoglobin, low systolic blood pressure and elevated BUN were significant correlates, again consistent with Smith and colleagues [9]. The findings for emergency surgery, where malignancy, NSAID use, elevated WBC and neutrophil counts, and lower albumin were significant, plausibly reflect coagulopathy, tissue invasion and tumour-related obstruction in malignancy-associated bleeding, together with the systemic inflammatory response of massive haemorrhage. For ICU admission, our results echo those of Tapaskar and colleagues [21], who linked advanced age, low haemoglobin and albumin, and high creatinine to ICU need; we additionally found malignancy, low-molecular-weight heparin and steroid use, hypotension, tachycardia, high INR and thrombocytopenia to be significantly associated with ICU admission.

The central finding of our study is that the Oakland Score was significantly associated with all of the adverse outcomes examined. This is consistent with the original validation by Oakland and colleagues [1], in whose work the score outperformed other LGIB scoring systems for predicting transfusion, rebleeding and readmission, and with Tapaskar and colleagues [21], who found the Oakland Score to have the highest accuracy for predicting severe bleeding. Li and colleagues [13] similarly reported the Oakland Score to be among the most accurate systems for predicting transfusion and adverse outcomes, and the systematic review and meta-analysis by Almaghrabi and colleagues [22] identified it as the best-performing tool for predicting safe discharge, severe bleeding and transfusion need. In the original study, the AUC for safe discharge was 0.87, with an AUC of 0.90 for RBC requirement, and a threshold of ≤8 predicted safe discharge with 98.4% sensitivity [1]. In our cohort the strong associations between the Oakland Score and RBC, platelet and FFP transfusion (all *p* < 0.001) reinforce its utility as a predictor of transfusion requirement.

The multivariable analyses provide important context for interpreting the Oakland Score’s prognostic role. Although the score was significantly associated with mortality and ICU admission on univariate analysis, it did not retain independent significance after adjustment for malignancy, serum albumin and BUN. This finding is consistent with the score’s primary design purpose (supporting safe-discharge decisions rather than predicting in-hospital mortality), and highlights that nutritional status (albumin) and tumour burden are the dominant independent determinants of fatal outcomes in this high-acuity cohort, in keeping with prior multivariate analyses of LGIB outcomes [13,21]. Notably, the Oakland Score was the only independently significant continuous predictor of one-month rebleeding, alongside malignancy, confirming that it adds clinically meaningful prognostic information for early rebleeding risk stratification beyond individual comorbidities [22]. Taken together, these multivariable results suggest that the Oakland Score is most valuable as part of an integrated clinical assessment incorporating albumin, malignancy status and renal function, rather than as a standalone mortality predictor.

On ROC analysis the Oakland Score discriminated best for ICU admission (AUC 0.754) and mortality (AUC 0.706), with high sensitivity (91.9% and 83.3% respectively), indicating that the score is particularly effective at identifying patients at risk of these severe outcomes, which carry substantial attributable mortality and prolonged intensive care stay [23]. Discrimination was moderate for one-month rebleeding and emergency surgery and weak for one-year rebleeding, suggesting that the score is better suited to identifying acute, in-hospital risk than to predicting longer-term rebleeding.

This study has several limitations. It was a single-centre, retrospective study, which constrains the generalisability of the findings and introduces the potential for selection and information bias. Furthermore, because only patients referred to the gastroenterology department from the emergency department were included, milder cases manageable on an outpatient basis were not captured, which may have inflated estimates of transfusion need, ICU admission and mortality relative to the broader LGIB population. Furthermore, the study did not include a direct comparison with other validated LGIB risk stratification tools such as the NOBLADS or Birmingham scores, which limits conclusions about the relative performance of the Oakland Score. Several variables required by those instruments were not systematically recorded in this retrospective dataset. Our findings should be interpreted in light of that evidence base. In addition, although lower gastrointestinal bleeding may originate from small-bowel lesions distal to the ligament of Treitz, dedicated small-bowel investigations, such as capsule endoscopy or device-assisted enteroscopy, were not routinely performed in this retrospective cohort. Furthermore, because the primary objective of this study was to evaluate the prognostic performance of the Oakland Score in a broad cohort of patients with lower gastrointestinal bleeding rather than to investigate diverticular bleeding specifically, detailed information regarding the anatomical location of colonic diverticula (right-sided versus left-sided) was not systematically collected or analysed. Therefore, the potential impact of diverticular location on clinical outcomes could not be assessed.

## 5. Conclusions

This study evaluated the performance of the Oakland Score in predicting clinical prognosis among patients presenting with LGIB to a tertiary referral centre. The score showed statistically significant associations with mortality, rebleeding, ICU admission, the need for surgery and blood-product replacement, supporting its role as a prognostic instrument in LGIB. The Oakland Score was particularly sensitive for predicting mortality and ICU admission, while its predictive power for some outcomes, notably longer-term rebleeding, was more limited. Higher scores were also associated with a greater need for blood-product replacement, suggesting that the score may serve as a meaningful indicator of transfusion requirement. Demographic and clinical data confirmed that LGIB occurs predominantly in older patients with frequent cardiovascular and metabolic comorbidities and malignancy, factors associated with longer hospital stay, greater ICU need and higher mortality. Consistent with the literature, reductions in haemoglobin and albumin and deterioration in vital signs were significantly associated with adverse outcomes. In conclusion, the Oakland Score provides a supportive method for risk stratification and management planning in patients with LGIB, but should be applied alongside the patient’s overall clinical status and other prognostic indicators. Larger, prospective, multi-centre studies are needed to more firmly establish its validity and effectiveness in routine clinical practice.

## Figures and Tables

**Table 1:** Demographic and clinical characteristics of the study population (*n* = 890).

Variable	Category	*n* (%)
Age (years), mean ± SD		69.8 ± 15.6
Sex	Female	449 (50.4)
	Male	441 (49.6)
Syncope/pre-syncope	Present	47 (5.3)
Hypertension	Present	500 (56.2)
Diabetes mellitus	Present	223 (25.1)
Coronary artery disease	Present	257 (28.9)
Cerebrovascular accident	Present	82 (9.2)
Inflammatory bowel disease	Present	37 (4.2)
Malignancy	Present	203 (22.8)
Cirrhosis	Present	30 (3.4)
Acetylsalicylic acid	User	257 (28.9)
Clopidogrel	User	121 (13.6)
Warfarin	User	72 (8.1)
Direct oral anticoagulant	User	82 (9.2)
Low-molecular-weight heparin	User	50 (5.6)
NSAID	User	113 (12.7)
Steroid	User	56 (6.3)

SD, standard deviation; NSAID, non-steroidal anti-inflammatory drug.

**Table 2:** Laboratory parameters, vital signs and Oakland Score.

Parameter	Mean ± SD	Median (Min–Max)
Lowest pre-colonoscopy Hb (g/dL)	8.95 ± 2.52	8.7 (2.7–16.1)
Lowest Hb in first 24 h (g/dL)	9.16 ± 2.56	9.0 (2.7–16.1)
Platelet (×10^3^/µL)	242 ± 113.7	226 (4–1032)
WBC (×10^3^/µL)	9.29 ± 4.95	8.2 (0.5–51.5)
RDW (%)	16.5 ± 3.29	15.6 (12.1–37.4)
INR	1.36 ± 1.13	1.03 (0.77–7.0)
BUN (mg/dL)	29 ± 21.76	22.3 (0.83–173.8)
Creatinine (mg/dL)	1.19 ± 0.9	0.94 (0.06–7.88)
Albumin (g/dL)	3.34 ± 0.6	3.37 (1.58–5.12)
Heart rate (beats/min)	87.8 ± 17	85 (45–179)
Systolic BP (mmHg)	127.3 ± 24	126 (53–220)
Diastolic BP (mmHg)	75.4 ± 15.2	75 (33–138)
Oakland Score	22.28 ± 6.15	23 (6–34)

Hb, haemoglobin; WBC, white blood cell; RDW, red cell distribution width; INR, international normalised ratio; BUN, blood urea nitrogen; BP, blood pressure; SD, standard deviation.

**Table 3:** Colonoscopic aetiology of lower gastrointestinal bleeding.

Aetiology	*n*	%
Diverticular disease	223	25.1
Inflammatory causes	189	21.2
Neoplasia	177	19.9
Anorectal disease	124	13.9
Angiodysplasia	98	11.0
Other	40	4.4
Post-polypectomy bleeding	23	2.6
Anastomotic bleeding	16	1.8

**Table 4:** Prognostic outcomes in patients followed for lower gastrointestinal bleeding (*n* = 890).

Outcome	*n*	%
RBC transfusion required	470	52.8
≤3 units (of those transfused)	282	60.0
>3 units (of those transfused)	188	40.0
Platelet transfusion required	41	4.61
FFP transfusion required	158	17.75
Rebleeding within 1 month	64	7.2
Rebleeding within 1 year	162	18.2
Hospital admission	625	70.2
ICU admission	37	4.15
Surgery	89	10.0
Emergency surgery	43	4.8
Mortality	54	6.1

RBC, red blood cell; FFP, fresh frozen plasma; ICU, intensive care unit.

**Table 5:** Selected factors significantly associated with mortality.

Variable	Survivors (*n* = 836)	Deaths (*n* = 54)	*p*
Age (years)	69.5 ± 15.7	75.6 ± 12.8	0.005
Malignancy, *n* (%)	172 (84.7)	31 (15.3)	<0.001
Cirrhosis, *n* (%)	25 (83.3)	5 (16.7)	0.030
LMWH use, *n* (%)	42 (84)	8 (16)	0.008
Steroid use, *n* (%)	46 (82.1)	10 (17.9)	0.001
Heart rate (beats/min)	87.3 ± 16.8	96.1 ± 19.0	<0.001
Systolic BP (mmHg)	128.1 ± 23.7	115.8 ± 26.3	<0.001
Lowest pre-colonoscopy Hb (g/dL)	9.03 ± 2.52	7.82 ± 2.27	<0.001
Platelet (×10^3^/µL)	245.8 ± 113.2	194.6 ± 111.6	0.001
BUN (mg/dL)	27.9 ± 20.5	47.5 ± 30.6	<0.001
Albumin (g/dL)	3.38 ± 0.57	2.73 ± 0.67	<0.001
LDH (U/L)	217.5 ± 193.7	430 ± 406.2	0.002
Oakland Score	22.03 ± 6.13	26.15 ± 5.03	<0.001

BP, blood pressure; Hb, haemoglobin; BUN, blood urea nitrogen; LDH, lactate dehydrogenase; LMWH, low-molecular-weight heparin. Values are mean ± SD unless otherwise stated.

**Table 6:** Association between the Oakland Score and blood-product replacement requirements.

Requirement	*n* (%)	Oakland Score (Mean ± SD)	*p*
RBC—No	420 (47.19)	17.48 ± 4.62	<0.001
RBC—Yes	470 (52.81)	26.57 ± 3.68	
Platelet—No	849 (95.39)	22.04 ± 6.14	<0.001
Platelet—Yes	41 (4.61)	27.17 ± 3.81	
FFP—No	732 (82.24)	21.30 ± 6.03	<0.001
FFP—Yes	158 (17.76)	26.81 ± 4.42	

RBC, red blood cell; FFP, fresh frozen plasma; SD, standard deviation.

**Table 7:** Multivariable binary logistic regression: independent predictors of adverse outcomes in acute lower gastrointestinal bleeding.

**A. Mortality**						
**Variable**	**B**	**SE**	**Wald χ^2^**	**OR**	**95% CI**	***p***
Oakland Score	0.028	0.038	0.535	1.028	0.954–1.108	0.465
**Malignancy**	**1.475**	**0.359**	**16.899**	**4.370**	**2.163–8.828**	<0.001 *
Cirrhosis	0.291	0.604	0.232	1.337	0.409–4.370	0.630
LMWH	0.506	0.575	0.775	1.658	0.538–5.115	0.379
Steroid	0.394	0.514	0.587	1.482	0.541–4.061	0.444
**BUN**	**0.018**	**0.006**	**8.199**	**1.018**	**1.006–1.031**	0.004 *
**Albumin**	**−** **1.537**	**0.345**	**19.899**	**0.215**	**0.109–0.422**	<0.001 *
Platelet	−0.002	0.002	1.670	0.998	0.995–1.001	0.196
INR	−0.051	0.185	0.075	0.951	0.661–1.367	0.785
Model χ^2^(9) = 90.269, *p* ≤ 0.001; Nagelkerke R^2^ = 0.3136; Hosmer–Lemeshow χ^2^(8) = 8.167, *p* = 0.4173.						
**B. ICU Admission**						
**Variable**	**B**	**SE**	**Wald χ^2^**	**OR**	**95% CI**	***p***
Oakland Score	0.097	0.050	3.796	1.101	0.999–1.214	0.051
**Malignancy**	**1.128**	**0.412**	**7.501**	**3.088**	**1.378–6.922**	0.006 *
**LMWH**	**1.384**	**0.567**	**5.951**	**3.991**	**1.313–12.137**	0.015 *
Steroid	0.675	0.559	1.456	1.964	0.656–5.879	0.228
BUN	−0.001	0.008	0.016	0.999	0.984–1.014	0.900
**Albumin**	**−** **1.734**	**0.387**	**20.066**	**0.177**	**0.083–0.377**	<0.001 *
Platelet	−0.001	0.002	0.652	0.999	0.995–1.002	0.419
INR	0.050	0.191	0.068	1.051	0.723–1.528	0.794
Model χ^2^(8) = 66.316, *p* ≤ 0.001; Nagelkerke R^2^ = 0.2903; Hosmer–Lemeshow χ^2^(8) = 14.946, *p* = 0.0602.						
**C. One-Month Rebleeding**						
**Variable**	**B**	**SE**	**Wald χ^2^**	**OR**	**95% CI**	***p***
**Oakland Score**	**0.079**	**0.024**	**10.826**	**1.082**	**1.032–1.133**	0.001 *
**Malignancy**	**0.599**	**0.277**	**4.660**	**1.820**	**1.057–3.136**	0.031 *
Model χ^2^(2) = 17.978, *p* ≤ 0.001; Nagelkerke R^2^ = 0.0495; Hosmer–Lemeshow χ^2^(8) = 13.356, *p* = 0.1002.						
**D. Emergency Surgery**						
**Variable**	**B**	**SE**	**Wald χ^2^**	**OR**	**95% CI**	***p***
Oakland Score	0.011	0.032	0.121	1.011	0.949–1.077	0.728
**Malignancy**	**0.794**	**0.356**	**4.961**	**2.212**	**1.100–4.448**	0.026 *
**NSAID**	**1.055**	**0.393**	**7.210**	**2.871**	**1.330–6.199**	0.007 *
WBC	0.033	0.027	1.490	1.033	0.980–1.089	0.222
**Albumin**	**−0.729**	**0.299**	**5.928**	**0.482**	**0.268–0.867**	0.015 *
Model χ^2^(5) = 23.176, *p* ≤ 0.001; Nagelkerke R^2^ = 0.0900; Hosmer–Lemeshow χ^2^(8) = 8.762, *p* = 0.3628.						

B, regression coefficient; SE, standard error; Wald, Wald chi-square statistic; OR, odds ratio; CI, confidence interval. LMWH, low-molecular-weight heparin; BUN, blood urea nitrogen; NSAID, non-steroidal anti-inflammatory drug; WBC, white blood cell count. * *p* < 0.05. Bold type denotes statistically significant variables. The Oakland Score was modelled with clinical/laboratory factors not encoded in the score to avoid multicollinearity. Hosmer–Lemeshow goodness-of-fit test *p* > 0.05 for all models, indicating adequate calibration.

## Data Availability

The datasets used and/or analysed in this study are available upon reasonable request from the corresponding author.
